# *Interleukin-2* and *Interferon-Gamma* Single Nucleotide Polymorphisms in Iranian Patients with Chronic Heart Failure

**Published:** 2018

**Authors:** Mohammad Jafar Mahmoudi, Sara Harsini, Elham Farhadi, Mona Hedayat, Mohammad Taghvaei, Maryam Mahmoudi, Maryam Sadr, Nilufar Esfahanian, Ebrahim Nematipour, Keramat Nourijelyani, Ali Akbar Amirzargar, Nima Rezaei

**Affiliations:** 1. Division of Cardiology, Department of Internal Medicine, Faculty of Medicine, Tehran University of Medical Sciences, Tehran, Iran; 2. Molecular Immunology Research Center, Tehran University of Medical Sciences, Tehran, Iran; 3. Department of Hematology, Faculty of Allied Medical Science, Iran University of Medical Sciences, Tehran, Iran; 4. Division of Immunology, Boston Children’s Hospital, Harvard Medical School, Boston, MA, USA; 5. Faculty of Nutrition and Dietetics, Tehran University of Medical Sciences, Tehran, Iran; 6. Tehran Heart Center, Tehran University of Medical Sciences, Tehran, Iran; 7. Department of Epidemiology and Biostatistics, Faculty of Public Health, Tehran University of Medical Sciences, Tehran, Iran; 8. Department of Immunology, Faculty of Medicine, Tehran University of Medical Sciences, Tehran, Iran; 9. Research Center for Immunodeficiencies, Children’s Medical Center, Tehran University of Medical Sciences, Tehran, Iran; 10. Network of Immunity in Infection, Malignancy and Autoimmunity (NIIMA), Universal Scientific Education and Research Network (USERN), Tehran, Iran

**Keywords:** Heart failure, Interferon-gamma, Interleukin-2, Single nucleotide polymorphism

## Abstract

**Background:**

Inflammatory cytokines have been known to be associated with Chronic Heart Failure (CHF). Given the importance of cytokines in the context of the failing heart, the prevalence of Interleukin-2 (IL-2) and Interferon-gamma (IFN-γ) polymorphisms was studied in patients with CHF due to ischemic heart disease in a case-control study.

**Methods:**

Fifty-six Iranian patients with CHF were enrolled in this study as the case group and compared with 139 healthy subjects, using polymerase chain reaction with sequence-specific primers method, so as to determine the frequency of alleles, genotypes and haplotypes of *IFN-γ* (+874 A/T) and *IL-2* (-330 G/T, +166 G/T) SNPs.

**Results:**

The GG genotype at *IL-2* -330 in patients with CHF was significantly over-represented in comparison with the control group (p=0.013). Such a positive genotypic association was also observed for *IL-2* +166/TT (p=0.022). Meanwhile, the GT genotype frequency at *IL-2* -330/GT in the patient group was significantly lower than the one in healthy controls (p=0.049). No significant association was detected between the *IFN-γ* gene polymorphisms and individuals’ susceptibility to CHF.

**Conclusion:**

Certain genotypes in *IL-2* gene were overrepresented in patients with CHF, which could render individuals more vulnerable to this disease.

## Introduction

Chronic Heart Failure (CHF) is a serious clinical condition, characterized by impaired contractile function and progressive ventricular dilation 1. As with any other major health issue, CHF greatly influences the quality of life of patients with this condition; therefore, it stands to reason that introduction of promising future genetic markers which could affect individual proneness to this disease, seems essential to initiate therapy in advance.

Various etiologies such as hypertension, coronary artery disease and infection, result in heart failure. Different heart failure models indicate the roles of cytokines, as major actors in different immune mechanisms, in the aforementioned etiologies leading to CHF 2–4. Interleukin-2 (IL-2) is one such proinflammatory cytokine, which induces proliferation of T cells 5. The other cytokine of this category is Interferon-gamma (IFN-γ), which is mainly produced by Natural Killer (NK) cells and T cells. This cytokine is known to be associated with T helper 1 (Th1) responses 6. Previous investigations have indicated the importance of these two inflammatory cytokines, IL-2 and IFN-γ, in the etiopathogenesis of various conditions such as atherosclerosis and ischemic and non-ischemic dilated cardiomyopathy, which could stand as the underlying cause of CHF development 7–9.

It has been postulated that Single Nucleotide Polymorphisms (SNPs) within coding and promoter sequences of cytokine genes could affect their secretion pattern 10,11. Numerous studies have been performed on cytokine gene polymorphisms in the context of various immunological disorders 12–20. However, due to the paucity of data regarding the contribution of cytokines’ gene polymorphisms in CHF susceptibility, achieving consensus seems impossible so far. To the best of our knowledge, this is the first study to explore certain IL-2 and IFN-γ gene polymorphisms in Iranian patients with CHF.

This study was conducted in a group of Iranian patients with CHF in order to assess the associations of SNPs in *IL-2* at positions −330 and +166 as well as *IFN-γ* at position +874 with the disease.

## Materials and Methods

### Subjects

Fifty-six Iranian patients with CHF (42 males, 14 females) with the mean age of 57.96±12.24 years were enrolled in this study. Diagnosis of CHF in patients was based on intensive history taking, thorough physical examination, electrocardiography and impaired Left Ventricular (LV) systolic function (LV ejection fraction ≤40%) and LV dilation (LV end-diastolic diameter >5.5 *cm*) on echocardiography. Subjects with recent myocardial infarction, malignancies, chronic lung disease and acute decompensated HF within 3 months before enrollment, were excluded from the study. Only those patients in stable clinical condition, who had received conventio¬nal medical therapy for at least 3 months, were enrolled in this study. Baseline demographic and clinical characteristics of patients with CHF, included in the current study, are depicted in table 1. One hundred and thirty nine unrelated healthy subjects (mean age 45.63±10.84; 100 men, 39 women) who were randomly selected from blood donors at Iranian blood transfusion organizations, were also selected as the control group 21. This study was approved by the Ethics Committee of Tehran University of Medical Sciences. Written informed consent was obtained from all participants prior to sampling.

**Table 1.** Baseline demographic and clinical characteristics of patients with CHF

**Variables**	**N (%)**
**Age (year)±SD**	57.96±12.24
**Sex (male) (%)**	43 (75.4%)
**Hypertension**	21 (36.8%)
**Diabetes**	19 (36.8%)
**Dyslipidemia**	22 (38.6%)
**Obesity**	8 (14%)
**History of smoking**	
Current smoker	25 (43.9%)
Ex-smoker	4 (7%)
Non-smoker	28 (49.1%)
**History of ACS**	31 (54.4%)
**COPD**	4 (7%)
**Chronic kidney disease**	5 (8.8%)
**Liver disease**	2 (3.5%)
**Cerebrovascular accident**	1 (1.8%)
**History of CABG**	5 (8.8%)
**History of PCI**	4 (7%)
**NYHAA classification**	
**I**	15 (26.3%)
**II**	18 (31.6%)
**III**	15 (26.3%)
**IV**	9 (15.8%)

COPD, Chronic Obstructive Pulmonary Disease; CABG, Coronary Rrtery Bypass Grafting; PCI, Percutaneous Coronary Intervention.

### Genotyping

After DNA extraction from the peripheral blood leukocytes using the “salting out” technique 22, the polymerase chain reaction, with sequence-specific primers (PCR-SSP assay kit; Heidelberg University, Germany) was employed for cytokine gene typing 21. Briefly, amplification of the extracted gene was performed by a Techne Flexigene thermal cycler (Roche) under the following conditions: initial denaturation at 94°*C* for 2 *min*; denaturation at 94°*C* for 10 *s*; annealing+extension at 65°*C* for 1 *min* (10 cycles); denaturation at 94°*C* for 10 *s*; annealing at 61°*C* for 50 *s*; and extension at 72°*C* for 30 *s* (20 cycles). Subsequently, the availability of the Polymerase Chain Reaction (PCR) products was assessed using 2% agarose gel electrophoresis. Thereafter, the gel was placed on an Ultraviolet (UV) transilluminator, and a picture was taken for analysis and documentation. The frequencies of alleles, genotypes and haplotypes of *IL-2* (G/T at −330 and +166) and *IFN-γ* (A/T at −+874) were counted.

### Statistical analysis

Allele, genotype and haplotype frequencies were calculated by direct gene counting. In order to test the Hardy-Weinberg equilibrium, the frequencies of various genotypes were compared using the chi square test. The odds ratios and 95% Confidence Intervals (CI) were estimated for each allele, genotype and haplotype. A p-value of less than 0.05 was considered significant.

## Results

### Alleles, genotype and haplotype frequencies

The allelic and genotype frequencies in patients with CHF and healthy controls are depicted in table 2. The GG genotype at IL-2 -330 in patients with CHF was significantly increased in comparison with the control group [p=0.013, OR=3.56, 95%CI: 1.32–9.57]. Such a positive genotypic association was also observed for IL-2 +166/TT [p=0.022, OR=6.72, 95% CI: 1.26–35.71]. Meanwhile, the GT genotype frequency at IL-2 -330 in the patient group was significantly lower than the one in healthy controls [p=0.049, OR=0.51, 95%CI: 0.27–0.97]. On the other hand, no significant association was found between the *IFN-γ* gene polymorphisms at +874 position and individuals’ vulnerability to CHF.

**Table 2.** *IL-2* and *IFN-γ* allele and genotype polymorphisms in Iranian patients with CHF and controls

**Cytokine**	**Position**	**Alleles/Genotypes**	**Controls (n=139) N (%)**	**Patients (n=56) N (%)**	**Odds Ratio (95% CI)**	**p-value**
IL-2	−330	G	110 (39.6)	49 (43.7)	1.19 (0.76–1.85)	0.495
		T	168 (60.4)	63 (56.3)		
		GG	8 (5.8)	10 (17.9)	3.56 (1.32–9.57)	0.013
		GT	94 (67.6)	29 (51.8)	0.51 (0.27–0.97)	0.049
		TT	37 (26.6)	17 (30.3)	1.2 (0.61–2.38)	0.600
	+166	G	219 (78.8)	80 (71.4)	0.67 (0.41–1.11)	0.145
		T	59 (21.2)	32 (28.6)		
		GG	82 (59)	29 (51.8)	0.75 (0.4–1.39)	0.425
		GT	55 (39.6)	22 (39.3)	0.99 (0.52–1.86)	1
		TT	2 (1.4)	5 (9)	6.72 (1.26–35.71)	0.022

			N=138	N=51		
IFN-γ	+874	A	140 (50.7)	49 (48)	0.89 (0.57–1.41)	0.728
		T	136 (49.3)	53 (52)		
		AA	43 (31.2)	12 (23.5)	0.68 (0.32–1.43)	0.369
		AT	54 (39.1)	25 (49)	1.5 (0.78–2.86)	0.247
		TT	41 (29.7)	14 (27.5)	0.89 (0.44–1.83)	0.858

No significant differences were found between the two groups for GG, TG, TT and GT haplotypes at positions −330 and +166 of *IL-2* gene (Table 3).

**Table 3.** *IL-2* haplotype polymorphism in Iranian patients with CHF and controls

**Cytokine**	**Position**	**Haplotype**	**Controls (n=138) N (%)**	**Patients (n=56) N (%)**	**Odds ratio (95% CI)**	**p-value**
**IL-2**	−330, +166	GG	107 (38.8)	46 (41.1)	1.1 (0.7–1.72)	0.731
		TG	112 (40.6)	34 (30.3)	0.64 (0.4–1.02)	0.065
		TT	56 (20.3)	29 (25.9)	1.37 (0.82–2.3)	0.226
		GT	1 (0.3)	3 (2.7)	7.57 (0.78-73.56)	0.074

## Discussion

Several pieces of evidence have shown that inflammation is an important actor in cardiovascular diseases, including Left Ventricular Dysfunction (LVD) and subsequent heart failure, which constitutes an ultimate common pathway for a multitude of cardiac disorders 23,24. Recently, the potential role of circulating inflammatory markers, such as IL-2 and IFN-γ, as risk predictors of cardiovascular events has been a topic of intensive research 25. It has long been speculated that cytokine gene polymorphisms could affect their serum level, as discussed in advance. Therefore, considering the significance of inflammation in cardiac diseases, the present study was designed to examine a sample of Iranian patients with CHF for the SNPs in *IFN-γ* gene at position +874 as well as *IL-2* polymorphisms at positions −330 and +166.

IL-2 is a secretory cytokine generated by activated T lymphocytes, which induces T cells, B cells, and NK cells to proliferate and produce other cytokines 26. Reports have suggested IL-2 as a predictor of vascular disease 7. Recent studies have proposed that elevated levels of IL-2 indicate intensified T cell response to different antigens, which are assumed to be critical in the promotion of atherosclerosis 7,8. Previous studies have suggested the *IL-2* G allele at position −330 is associated with enhanced IL-2 expression. *IL-2* (−330) GG genotype is recognized as a polymorphism with an increased level of cytokine production following anti-CD3/CD28 stimulation of lymphocytes. However, the GT genotype at the same position is acknowledged as a genotype with an intermediate level of *IL-2* gene expression. *IL-2* (−330) TT genotype is also known to cause low IL-2 levels 27. Our statistical analysis of *IL-2* gene polymorphisms disclosed increased frequency of *IL-2* -330 GG genotype as well as *IL-2* +166 TT genotype in patient group, compared with control category, while *IL-2* -330 GT genotype was shown to be more frequent in healthy controls. Our results are consistent with the findings of a recent study conducted by Ding *et al* 28, which revealed the association of *IL-2*-330 GG genotype with increased risk of coronary artery disease. Their results also showed that subjects carrying *IL-2* -330 GG genotype had increased serum level of IL-2 in comparison with those with TG or TT genotypes 28.

IFN-γ is a proinflammatory cytokine produced by Th1 cells, which enhances the expression of MHC class I and class II molecules. An increment of IFN-γ-positive CD4 (+) T cells has been previously reported in patients with CHF 6. On the contrary, diminishment in IFN-γ serum levels has been described 29, in a group of patients with CHF secondary to ischemic and non-ischemic dilated cardiomyopathy. Several investigations have demonstrated the expression of IFN-γ in the immunological activation of atherosclerotic lesions from both clinical samples together within preclinical mouse atherosclerosis models 9,30. Among multitude of SNPs reported in *IFN-γ* gene, *IFN-γ* +874 A/T polymorphism, which maps to the putative Nuclear Factor-kB (NF-kB) binding site is known to enhance the expression of *IFN-γ* gene, where A mutant allele is present, while the presence of T allele is known to be involved in underexpression of *IFN-γ* 31. Multiple studies carried out in both humans and rodent models have investigated a proatherogenic role of IFN-γ 32–35. Garg *et al* 36 suggested a significant role of *IFN-γ* +874 T allele in the occurrence of coronary heart disease. In the current investigation, no association was found between polymorphisms in *IFN-γ* at position +874 and individual susceptibility to CHF.

This study has certain limitations that should be acknowledged. Firstly, our limitations to measure the serum levels of IL-2 and IFN-γ hindered evaluation of the relevance of gene variants in terms of cytokine levels in patients with CHF. Additionally, previous studies performed in this field have not resulted in a consensus regarding the aforementioned cytokines’ serum levels in patients with CHF, as some have reported elevated levels of IL-2 and IFN-γ in patients with CHF, while some others have suggested decreased levels of these cytokines in such patients. These contradictory results warrant further analysis of Il-2 and IFN-γ levels in CHF. Moreover, the relatively small number of our subjects could diminish the statistical power of our analysis.

## Conclusion

To conclude, it is suggested that certain single nucleotide polymorphisms in *IL-2* gene can affect the risk of developing CHF. These associations may help us define both predisposing and protective genetic markers with regard to CHF. However, in order to delineate the role of *IFN-γ* and *IL-2* genotypes in the pathogenesis of CHF and influence on IFN-γ and IL-2 production, further studies on cytokine gene polymorphisms in other populations, using larger sample size, are required.
